# Determinants of Hospital Pharmacists’ Job Satisfaction in Romanian Hospitals

**DOI:** 10.3390/pharmacy5040066

**Published:** 2017-12-11

**Authors:** Magdalena Iorga, Corina Dondaș, Camelia Soponaru, Ioan Antofie

**Affiliations:** 1 Department of Behavioral Sciences, University of Medicine and Pharmacy “Grigore T. Popa”, Iasi 700115, Romania; 2 Department of Career Counseling, University of Medicine and Pharmacy “Grigore T. Popa”, Iasi 700115, Romania; dondascorina@gmail.com; 3 Department of Psychology, University “Alexandru Ioan Cuza”, Iasi 700506, Romania; puzdriac@yahoo.com; 4 Department of Hospital Pharmacy, C.F. Hospital, Cluj-Napoca 599597, Romania; ioanantofie@ymail.com

**Keywords:** hospital pharmacists, job satisfaction, personality traits

## Abstract

*Aim*: The purpose of this study is to identify the level of job satisfaction among hospital pharmacists in Romania in relation to environmental, socio-demographic, and individual factors. *Material and Methods*: Seventy-eight hospital pharmacists were included in the research. The Job Satisfaction Scale was used to measure the level of satisfaction with their current jobs, and the TAS-20 was used to evaluate emotional experience and awareness. Additionally, 12 items were formulated in order to identify the reasons for dissatisfaction with jobs, such as budget, number of working hours, legislation, relationships with colleagues, hospital departments, or stakeholders. Data were analyzed using IBM SPSS Statistics version 23. *Results*: The analyses of the data revealed a low level of satisfaction regarding the pay–promotion subscale, a high level of satisfaction with the management–interpersonal relationship dimension, and a high level of satisfaction regarding the organization–communication subscale. Seventy-four percent of subjects are dissatisfied about the annual budget, and 86.3% are not at all satisfied with present legislation. *Conclusions*: These results are important for hospital pharmacists and hospital management in order to focus on health policies, management, and environmental issues, with the purpose of increasing the level of satisfaction among hospital pharmacists.

## 1. Introduction

Job satisfaction is an important factor for increasing a person’s involvement in the workplace, and also for motivation [1]. It represents a combination of positive or negative feelings that workers have towards their work, and the perceived relationships between a person’s expectations and the actual results. Satisfaction with work is closely linked to that individual’s behavior in the workplace [2,3,4,5].

The interest in studying job satisfaction has a two-fold purpose: Identifying the various factors that may increase the pharmacists’ workplace satisfaction, and, implicitly, patients’ satisfaction, as well as improving the quality of the services provided [6,7,8,9]. When compared to the general population, older data show that pharmacists may be slightly less satisfied with their specific jobs than the general population [7].

Over the decades, the profession of hospital pharmacist interacted closely with other medical health care professionals in hospitals; hence, the importance of pharmacists in a multidisciplinary team has increased [8]. As part of a team providing health services, pharmacists are involved in accomplishing various tasks and in making decisions that have an impact on a patient’s quality of life. Thus, job satisfaction level may actively influence employees’ motivation to get involved in hospital activities [10,11,12].

Kerschen et al. [13] reported that hospital pharmacists obtained mean satisfaction scores, and some studies identified that the primary factor in employee retention is employee job satisfaction [14,15]. Among professionals in the same pharmaceutical department, pharmacists appeared to be more satisfied than support personnel [7]. Job positioning and skill use repeatedly appeared to be related to pharmacist job satisfaction [15].

Factors that influence job satisfaction are related to a burnout work pattern [16], work environment, external pressures (commercials), and sector of practice [17,18,19]. Some studies have shown that hospital pharmacists are more satisfied when compared to community pharmacists, who are more pressured by the number of working hours, by a poor relationship with physicians, or by the lack of promotion opportunities [20]. Job dissatisfaction impacts job performance, resulting in an increased number of errors [21,22].

The levels of job satisfaction among hospital pharmacists was identified by studies revealing different scores. For example, 77% of hospital pharmacists were satisfied with their jobs in Australia [23], 67% in India [24], and 67.3% in the United States of America (USA) [25].

Previous studies showed that socio-demographic factors have an influence on pharmacist job satisfaction. For example, a study performed by Majd et al. [26] showed that younger pharmacists were significantly less satisfied with their incomes compared to older pharmacists, while other studies [27] reported that men were less satisfied compared to women. Additionally, there are studies that found a positive relationship between job factors and a hospital pharmacist’s level of job satisfaction. Different studies showed that gender and job position influence overall job satisfaction to a significant degree [28]. When it comes to motivation factors, pharmacists ranked recognition, promotion, job satisfaction, job feedback, autonomy, and task significance among the most influential motivators to pharmacists. Pharmacists’ superiors considered financial rewards to be more important than non-financial incentives and benefits [29]. In a previous study [30] targeting forensic physicians, it was found that alexithymia is negatively correlated with one of the factors of job satisfaction, namely, organization and communication skills, and since alexithymia is metaphorically considered an “emotional numbness”, determination as to whether it has an influence on pharmacists, as well as whether it affects their job satisfaction level, is wanted.

Several studies have been conducted on hospital pharmacists’ satisfaction, but this topic has not been analyzed in Romania thus far. In Romania, private or public hospitals comprise hospital pharmacists and clinical pharmacists. The former administers the pharmacy department; they deal with the supply and delivery of medicines for hospital departments and of subsidized drugs. The latter, which consists of clinician pharmacists, must be assigned to a medical or surgical clinic: They advise physicians concerning doses and they contribute to the personalized medicine programs of all patients.

Six university pharmacy schools and two departments of pharmacy within medical schools, providing medico-pharmaceutical education, are accredited by the Romanian Ministry of Education. The standard curriculum is about five years in length, including six months of internship training, in country or abroad. From a legal point of view, Romania conforms to European Union (EU) directives regarding education and mutual recognition of diplomas for pharmacists [31,32].

The number of pharmacists (76.3 for 100,000 inhabitants) is lower in Romania, as compared to the European Union average of 82.8, but is similar to that of Eastern European countries [32,33]. There are approximately 5200 pharmacies in the country, and each is mandated to have at least one pharmacist. Each of the 450 hospitals has a pharmacy where one to three hospital pharmacists usually work [33]. The College of Pharmacists is the national association with which all of the pharmacists should register, as noted by specific legislation. One-hundred and sixty pharmacists that are working in hospitals are also registered in The National Association of Hospital Pharmacists in Romania (ANFSR), and their salaries are paid by hospital pharmacies. The right to free practice is granted by the College of Pharmacists, and pharmacists may follow specialized postgraduate training courses. 

The graduation process is assured by a residency exam in general pharmacy. However, in the period of 2004–2017, this exam was not organized. The only residency exams that were organized in the last 13 years were for clinical pharmacy and for laboratory pharmacy. Currently, hospital pharmacists (ANFSR), supported by the College of Pharmacists, are struggling to establish, with the Health Minister, the promotion of new laws for the benefit of hospital pharmacists, as well as the quality of pharmaceuticals in hospitals.

The annual budget that hospital pharmacists have to manage is limited by hospital decisions. A study analyzing the transition period and the impact on health policies in Romania found that health spending increased significantly between 2001 and 2007. This trend continued in the following years, due to the high level of expenditures for both hospital services and pharmaceuticals; for example, most of expenditures that were registered in 2005, for instance, were payments of debts pertaining to the previous year [34].

Thus, promotion and budget could be two main important sources of dissatisfaction among hospital pharmacists. The goal of this study is to evaluate the level of job satisfaction among hospital pharmacists in Romania, and to identify the influence of socio-demographic, environmental, or personality constructs, such as alexithymia on work satisfaction. The present study is the first one of its kind conducted in Romania, and its findings are important because they will join those obtained by studies conducted in other countries. The similarities and differences in the tasks of hospital pharmacists in different countries lead to diverse levels of satisfaction with work. Some of them are related to personality factors, but others are determined by national health policies or by the transition process. 

## 2. Material and Methods

The present research was part of a broader study that was approved by the National Association of Hospital Pharmacists in Romania (ANFSR). Seventy-eight subjects, out of 160 pharmacists registered with ANFSR (a rate of 48.75%), answered questionnaires. The subjects worked in public hospitals, in 20 out of the 42 counties in Romania (covering more than half of the country’s territory). Informed consent and a document including personal data were obtained before the questionnaires were completed. Subjects were informed about the confidentiality of personal data, as well as the research objectives. 

In order to identify problems related to job satisfaction among pharmacists working in public hospitals, and to evaluate the level of satisfaction with their jobs, the following instruments were applied:

(a) a fill-in form, including socio-demographic characteristics (age, gender, department, work experience in years, years of work at the present institution, quality of head pharmacist);
(b) an open-ended questionnaire with 12 items, regarding job-related opinions and regarding relationships with colleagues, staff, and stakeholders; and,
(c) two psychological tools to evaluate pharmacists’ level of alexithymia and the level of satisfaction with their jobs (Toronto Alexithymia Scale and Job Satisfaction Scale).

The Job Satisfaction Scale—JSS [35]—consists of 32 items representing three factors that were related to job satisfaction: Pay–promotions (14 items), management–interpersonal relationships (eight items), organization–communication (10 items), and overall job satisfaction. The questionnaire was adapted from the “Job Satisfaction Survey”, proposed by P.E. Spector in 1997, a tool with 36 items evaluating nine aspects of job satisfaction: Payment, promotion, supervision, secondary benefits, potential rewards, regulations, co-workers, the nature of work, and communication. The adapted version of this scale, containing 32 items, was applied to a population of 566 subjects with the following internal consistency coefficients: Payment and promotion, 0.820; leadership and interpersonal relationships, 0.760; and organizational and communication, 0.738; the Cronbach’s alpha coefficient for the total score was 0.872.

The Toronto Alexithymia Scale—TAS-20 [36] is a personality construct, called “emotional blindness”. It refers to trouble in identifying and describing emotions and the tendency to minimize emotional experience and focus attention externally. It was used for this research to identify the difficulties with emotional processing and emotional awareness. It is a questionnaire that demonstrates a good internal consistency (Cronbach’s alpha = 0.81) and test–retest reliability (0.77, *p* < 0.001), adequate levels of convergent, and concurrent validity in other studies [37]. The authors found it to be stable and replicable across clinical and nonclinical populations. In the present study, hospital pharmacists had to rate answers on a Likert–type scale (1 = strongly disagree to 5 = strongly agree). A total of 20 items targeted three domains: Difficulty describing feelings, difficulty identifying feelings, and externally oriented thinking. The total score for alexithymia was calculated as the sum of these three subscales. A score lower than or equal to 51 meant that subjects did not have alexithymia; a score between 52 and 60 was equal to borderline alexithymia, and subjects with a total score higher than or equal to 61 were characterized by alexithymia.

The obtained data were processed using IBM SPSS Statistics version 23 (IBM Japan, Tokyo, Japan). Mean and standard deviation were used for descriptive analyses of data, the *t*-test for independent samples, and one-way ANOVA for comparative analysis, in addition, Pearson and Spearman correlations were used to point out the relationships between variables.

## 3. Results and Discussions

### 3.1. Descriptive Analysis

Instruments: For the three dimensions of the JSS score, the Cronbach’s alpha scores were: 0.710 for pay–promotions, 0.679 for management–interpersonal relationships, and 0.796 for organization–communication. The Cronbach’s alpha total score for job satisfaction was 0.834. For TAS-20, Cronbach’s alpha scores obtained for all the three dimensions were: 0.438, 0.783, and 0.523, respectively. The total score for alexithymia was 0.738, suggesting a good internal consistency. The low score for Cronbach’s alpha concerning the first domain of TAS was probably due to two causes: The number of variables that are not normally distributed, and the existence of lower and higher scores that contribute to the medium level of the total score. For this reason, the results referring to this domain should be cautiously considered as conclusions.

Socio-demographic data: Seventy women (89.7%) and eight men (10.3%) were included in the research, with 59 (75.6%) being heads of hospital pharmaceutical departments (mean age 45.57 ± 10.12, with a minimum age of 25 and a maximum age of 61). In total, 47 pharmacists (60.3%) were married or in a relationship, and 52 of them (67.5%) had children; 31 subjects (39.7) reported as being single. The duration of experience in the pharmaceutical field in years is M = 19.07 ± 11.23 (with a minimum of one and a maximum of 37 years of work). The period of employment at their current jobs was 10.81 ± 9.78 (with a minimum of one and a maximum of 33 years), the number of working hours per week was 37.51 ± 3.96 (with a minimum of 35 and a maximum of 50 h/week).

Job-related data: Twelve items were formulated. Seven items focused on identifying the relationship between the pharmacist and other members of hospital staff (managers, medical directors, sales managers, pharmaceutical company representatives). They had to answer on a Likert-type of scale from 1 (never) to 5 (always). The frequency of answers to these items is presented in Table 1.

The most frequent conflictual relationships are those with staff working in the purchasing department, and with pharmaceutical company representatives. In what concerns their relationships with the latter, conflicts may be explained by the fact that medical warehouses no longer supply hospitals with medication if its unpaid bills exceed three to six months. The purchasing department is in charge of concluding framework agreements and subsequent contracts, and is also responsible for maintaining connections with medical warehouses. Therefore, it is the intermediary link between the hospital pharmacy and the medical warehouse; however, a pharmacist, in fact, has little power when it comes to accelerating the process of delivering medications to a patient. Therefore, the results may reflect the awareness of the pharmacist’s inability to categorically influence the process of delivering drugs to beneficiaries.

Five other items questioned hospital pharmacists’ opinions regarding: Item (8) current legislation; item (9) budget; item (10) working time; item (11) relationships with colleagues; and, item (12) reasons for conflicts with peers. Most hospital pharmacists (*N* = 63, 86.3%) declared that they were not at all satisfied with the present legislation. The responses to the item questioning the pharmacists’ opinions regarding working time revealed that 60 of them (83.3%) were satisfied with their working hours/week, and only 12 of them (16.7%) were not satisfied with their weekly schedules. When compared to other healthcare personnel working at a hospital, pharmacists are not frequently obligated to have extra working hours.

Hospital pharmacists were asked if they were satisfied with the relationships with their colleagues from the pharmaceutical department. Seventy-one subjects (95.9%) claimed that they were satisfied, and only three of them (4.1%) reported not being satisfied with the relationships with their colleagues. Among the reasons that were mentioned as causes of conflicts between pharmacists, the questioned subjects mentioned communication (*N* = 35, 52.2%), subordinated relationships (*N* = 8, 11.9%), job-related tasks (*N* = 20, 29.9%), and job description (*N* = 4, 6%).

Regarding pharmacists’ contentment with the annual budget, the frequency of their responses showed that only 19 (26%) were satisfied with the financial budget at their disposal, and 54 (74%) were discontent with it. The results showed that the most stringent problem at work was related to budget. In Romania, an allotted budget refers to the sum of money that is allocated by hospitals for medicine. A pharmacist has to manage this sum to ensure that a hospital has the minimum amount of medicines needed. Prior to the constitution of the Health Insurance House, funds for medicines used to come from a separate budget line; the money currently arrives in one large payment, which the manager distributes as they see fit, and purchasing medicine is not always a priority.

Psychological data: The result for TAS-20 (total score = 44.89) showed that pharmacists had a low level of alexithymia (*N* = 55, 74.3% non-alexithymia, *N* = 4, 18.9% with borderline alexithymia, and *N* = 5, 6.8% with alexithymia). Detailed results for all three dimensions, and for alexithymia and job satisfaction subscales, are presented in Table 2.

**Figure 1:**
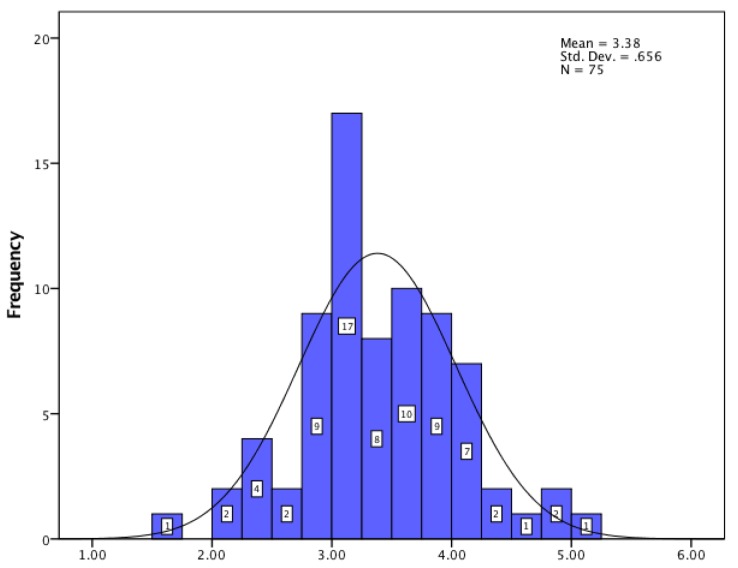
Payment and promotion.

**Figure 2:**
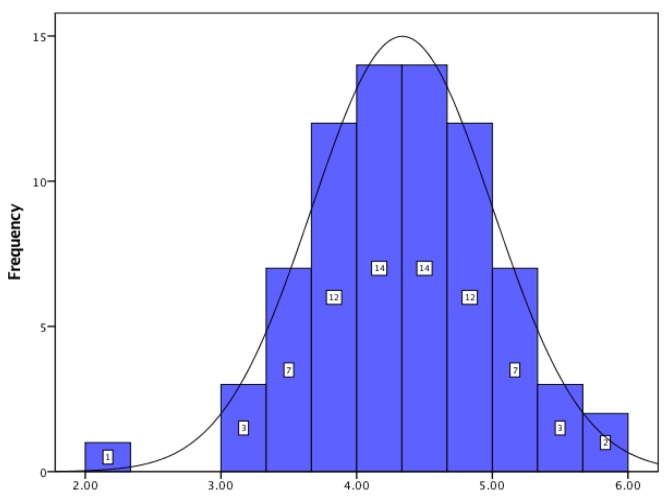
Management and interpersonal relationships.

**Figure 3:**
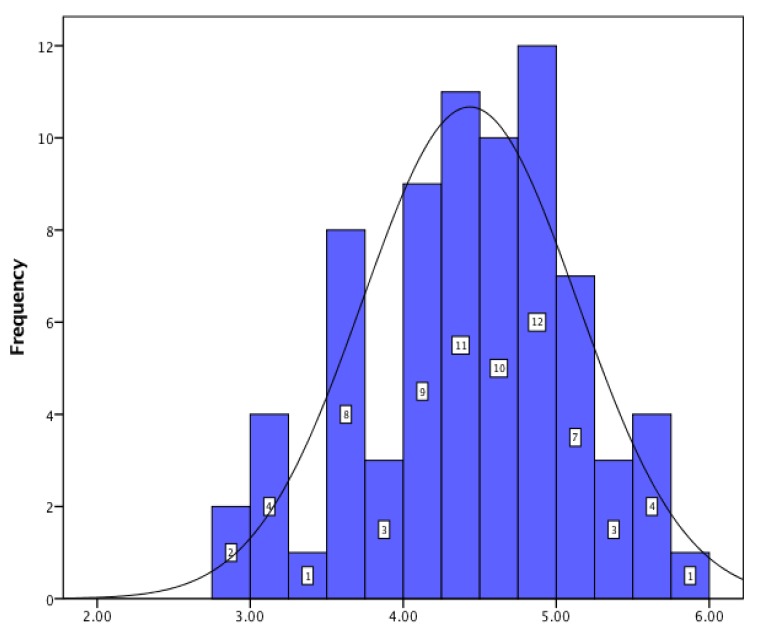
Communication and organization.

For JSS, the obtained data showed that, regarding pay–promotion, hospital pharmacists showed a low level of satisfaction; for management–interpersonal relationships the score proved a very high level of satisfaction; and for organization–communication, hospital pharmacists had a high level of satisfaction. The histograms for all three dimensions of job satisfaction are presented in Figure 1, Figure 2 and Figure 3.

The results that were obtained in the three subscales of job satisfaction emphasized the fact that the greatest level of discontent was related to promotion possibilities and pay level. In Romania, promotion is based on a medical residency exam and training. In general practice pharmacy, no residency exam has been organized since 2004, as was highlighted in the Introduction. Therefore, promotion occurs within the pharmacy, and the chances of getting a promotion are minimal. Nevertheless, upon analyzing an individual’s right to improve and develop at the workplace, we considered that the currently-implemented conditions of the hospital pharmacist position are in violation of the constitutional right to develop at work.

### 3.2. Correlation Analysis

In order to choose an appropriate statistical procedure, the normality of the data distribution with a sample Kolmogorov–Smirnov test was tested. For the alexithymia construct, it was found that two dimensions are not normally distributed (difficulty describing feelings and difficulty identifying feelings), while the third component (externally oriented thinking) and the total score of alexithymia had a normal score distribution. For JSS, all of the dimensions and total scores are normally distributed.

For the socio-demographic variables (age, total number of years in the pharmaceutical field, working years in the hospital, and the number of working hours per week), it was found that they are not normally distributed. Pearson correlations were used for the variables that were normally distributed, and, for variables that were not normally distributed, Spearman correlations were applied. The correlation analyses between variables that were considered were not, in general, statistically significant. The only significant correlations were negative, namely those between the management–interpersonal relationship dimensions (JSS), with externally-oriented thinking (R = −0.272 *, *p* = 0.021) and total score for alexithymia (R = −0.241 *, *p* = 0.042). The results showed that the more externally-oriented thinking a person/alexithymic is, the less satisfied they are regarding management–interpersonal relationships.

This third dimension of TAS-20 refers to an individual’s tendency to focus their attention externally. Management and interpersonal relationships refer to satisfaction with their relationships with colleagues and superiors, and to a tendency to avoid conflicts. It seems that a person who focuses on external events is more prone to being sensitive to them, and are less satisfied with events and relationships. This explanation is also valid for the total alexithymia score.

No correlations were identified between age, experience, number of working hours, or the other two subscales of TAS-20, and satisfaction with the pay–promotion or organization–communication sub-dimension of the JSS. The findings of this study are at odds with the results obtained by other studies (age and sex did not have an impact on job satisfaction of pharmacists) [26,27,28].

### 3.3. Comparative Analysis

In order to perform a comparative analysis, a *t*-test for independent variables was used and one-way ANOVA was used for the variables with more than two levels. The comparative analysis considering the independent variables of age (25–40, 41–55, 56–65 years old), level (pharmacist, specialist, or hospital primary pharmacist), administrative tasks (pharmacist or head pharmacist), satisfaction with the annual budget, colleagues, legislation, or work time revealed no significant differences.

The only significant statistical difference that was obtained was between pharmacists that were satisfied with the annual budget and those who were not. Subjects who reported being more satisfied with the financial budget were more satisfied with management–interpersonal relationships (Msatisfied = 4.53, Mdissatisfied = 4.19; *t* (68) = 2.037, *p* = 0.046) and organization–communication (Msatisfied = 4.63, Mdissatisfied = 4.26; *t* (68) = 2.071, *p* = 0.042), and with their job—total job satisfaction (Msatisfied = 4.07, Mdissatisfied = 3.80; *t* (68) = 2.025, *p* = 0.047). No significant difference was identified when considering the levels of alexithymia and job satisfaction.

The present research showed that the low level of satisfaction was related to the pay and promotion dimension. External motivation factors influence job satisfaction, given that some studies have found recognition, promotion, and pay to be important factors for work satisfaction [27]. These findings were congruent with results from the literature. In a study by Popa and Bazgan, two types of factors were identified as being responsible for the level of job satisfaction: Extrinsic factors deriving from the organizational context (salary, policies practiced in the organization, working conditions, autonomy and control, job security, interpersonal relations), and intrinsic factors, related to personal experience and to the relationship with the work environment (recognition by others, responsibility for own work and others’ work, advancement, personal fulfilment) [38].

The findings of this study showed that some variables, such as age, level (pharmacist, specialist, or hospital primary pharmacist), administrative tasks (pharmacist or head pharmacist), satisfaction with the annual budget, the relationship with their colleagues, legislation, or work time revealed no significant differences.

The results were consistent with the findings of other studies, which found that gender, job positions, education levels, the size and location of hospitals, and work experience were not significant factors in determining job satisfaction. As some studies have shown, job-related predictors of job satisfaction are skill use (as the most important factor in evaluating their ideal job) and recognition [37,39].

Payment and promotion should be related to a method of acknowledgement, as well as to the possibility of professional development and promotion, which is not encouraged by the present Romanian legislation. Recognition is also related to the job tasks of hospital pharmacists in Romania: Hospital pharmacists are not part of the medical team, and they are not involved in medical decisions. Access to a patient’s medical data chart is not always allowed for pharmacists, and only in a small number of medical cases do physicians seek their advice regarding drug administration. In this situation, a hospital pharmacist cannot determine whether a medical treatment is appropriate for the needs or conditions of a patient.

Hospital pharmacists are not always involved in the elaboration of medical guidelines; their involvement in therapeutic acts being limited. The restricted participation in the provision of healthcare has, on the other hand, a bright side. Hospital pharmacists are not held liable for professional errors, ethical dilemmas, or malpractice issues. When compared to other medical specialties in the country, hospital pharmacists have higher scores for job satisfaction than forensic physicians or obstetrics and gynecological physicians in the country [30].

### 3.4. Strengths and Limitations of the Study

The strength of the present study is provided by the results referring to a medical specialty scarcely featured in the scientific literature. Results of studies targeting hospital pharmacists’ job satisfaction are missing in Romania, and the present study is the first that is developed in the country. The results are representative, when considering the fact that the number of hospital pharmacists working in public institutions is relatively small.

The first limitation is due to the small number of male subjects, and a significant comparative analysis was not statistically relevant; the second limitation is related to national policies and legislation that is applied in this medical field, which have an important impact on the level of satisfaction among hospital pharmacists in Romania. Finally, another limitation is due to the poor Cronbach’s alpha score for the first domain of TAS-20, which is why the results should be considered cautiously.

## 4. Conclusions

Hospital pharmacists in Romania obtained a low level of satisfaction with respect to payment and promotion dimensions, as well as a very high level of satisfaction for the management–interpersonal dimension, and a high level of satisfaction for the organization–communication dimension of job satisfaction. Most hospital pharmacists are discontent regarding the annual budget, the present legislation, and the relationships with staff working in the purchasing department and pharmaceutical company representatives. Since individual characteristics or alexithymia do not influence the level of job satisfaction, the results are important for health policy-makers in order for them to find ways to increase job satisfaction among hospital pharmacists in Romania, by focusing on professional issues and environmental factors, as well as on health policies.

## Figures and Tables

**Table 1:** The frequency of answers for items I1–I7.

	Items Do You Have Conflicts with …	Never	Sometimes	Often	Most of the Time	Always
I1	The purchasing department	38.7%	46.7%	8%	6.7%	0%
I2	Hospital general manager	55.3%	34.2%	6.6%	2.6%	1.3%
I3	Hospital medical manager	66.7%	32%	0%	1.3%	0%
I4	Heads of clinical departments	48%	49.3%	2.7%	0%	0%
I5	Physicians	50.7%	46.7%	1.3%	1.3%	0%
I6	Pharmaceutical companies	28.9%	60.5%	7.9%	1.3%	1.3%
I7	Colleagues at the pharmacy	79.2%	18.1%	1.4%	1.4%	0%

**Table 2:** Results for Job Satisfaction Scale and Toronto Alexithymia Scale.

Instruments	Domains	Mean ± Standard Deviation
*JSS*	Payment–promotion	3.38 ± 0.65
	Management–interpersonal relationship	4.33 ± 0.66
	Organization–communication	4.41 ± 0.70
	Total score	3.92 ± 0.55
*TAS-20*	Difficulty describing feelings	12.97 ± 5.48
	Difficulty identifying feelings	13.90 ± 5.18
	Externally oriented thinking	18.01 ± 4.27
	Total score	44.89 ± 11.56
